# Grave infarctus mésentérique par occlusion artérielle mésentérique supérieure chez un patient atteint de la maladie de Buerger

**DOI:** 10.11604/pamj.2014.19.322.5718

**Published:** 2014-11-26

**Authors:** Moulay Ibrahim Ratbi, Ghislain Yves Abissegue, Mohamed Tarchouli, Mohammed Tariq Tajedine

**Affiliations:** 1 Service de Chirurgie Viscérale, Hôpital Militaire d'Instruction Mohammed V, Rabat, Maroc; 2 Service de Chirurgie Vasculaire, Hôpital Militaire d'Instruction Mohammed V, Rabat, Maroc

**Keywords:** Maladie de Buerger, infarctus mésentérique, thrombose, artère mésentérique supérieure, Buerger disease, mesenteric infarction, thrombosis, superior mesenteric artery

## Abstract

La thromboangéite oblitérant ou maladie de Buerger, est une artérite inflammatoire non-artériosclérotique touchant classiquement les réseaux vasculaires périphériques des membres. Elle atteint principalement les hommes jeunes tabagiques et sans autres facteurs de risques cardiovasculaires. Les atteintes des artères digestives sont très rares et souvent fatales si elles ne sont pas évoquées et prises en charge précocement. Nous rapportons l'observation d'un jeune patient tabagique chronique qui s’était présenté aux urgences dans un tableau de péritonite aigue négligée due a un infarctus entero-mésentérique massif. L'origine était une ischémie mésentérique due à une thrombose de l'artère mésentérique supérieure. L’étude anatomopathologique avait objectivée une atteinte des artères digestive due à la maladie de Buerger.

## Introduction

La thromboangéite oblitérant ou maladie de Buerger, est une artérite inflammatoire non-artériosclérotique touchant classiquement les réseaux vasculaires périphériques des membres. Elle atteint principalement les hommes jeunes tabagiques et sans autres facteurs de risques cardiovasculaires. Les atteintes des artères digestives sont très rares et souvent fatales si elles ne sont pas évoquées et prises en charge précocement.

## Patient et observation

**Figure 1:**
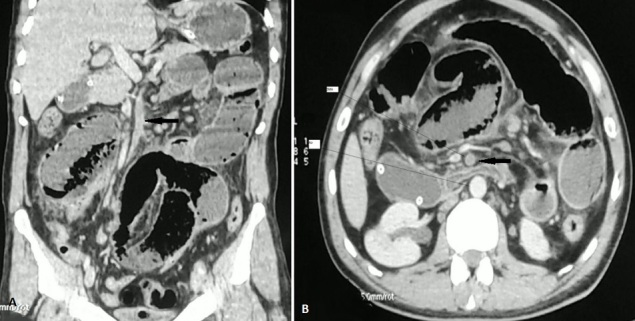
TDM Abdominale C+: paroi digestive très fine non rehaussée signant une ischémie d'origine artérielle. Défect endoluminale non rehaussée après injection: thrombus dans l'artère mésentérique supérieure (SMA) (flèche noire) A) reconstruction coronale; B) coupe axiale

Un patient de 37 ans s’était présenté aux urgences dans un tableau de douleurs abdominales et de vomissements évoluant depuis 2 jours. Il était connu tabagique chronique à 20 paquet/années. 12 mois au paravent, il avait consulté pour des claudications intermittentes du membre inférieur droit et gangrène sèche du gros orteil. Un bilan étiologique avait été effectué. A la biologie il ne présentait pas de dyslipidémie ni de syndrome inflammatoire et son bilan de coagulation était normal; les explorations cardiaques (électrocardiogramme et échocardiographie trans-oesophagienne) n'avaient trouvé aucune anomalie; un angioscanner des membres inférieurs (clichés non disponibles) objectivait une atteinte des axes jambiers qui avaient un aspect grêle sans sténose ni calcifications. Une atteinte due à la maladie de Buerger était retenue. Le patient subissait une amputation d'orteil, était mis sous antibiotique, antiagrégant plaquettaire, associé à l'arrêt du tabac. Au bout de 2 mois de traitement on constatait l'amélioration de son périmètre de marche et la cicatrisation des lésions. Le patient était par la suite perdu de vue. L'examen clinque à son admission trouvait un patient fébrile à 40 °C; tachycarde à 110 battement/min; polypnéique à 25 cycle/min; une tension artérielle (TA) à 110 / 60 mmHg); une saturation en oxygène (SpO2) à 98%. Son abdomen était sensible avec une défense généralisée. Ses pouls fémoraux et poplité étaient présents mais es pouls tibiaux postérieur et pédieux étaient abolis bilatéralement. Son taux d'hémoglobine était de 13g/dl, le nombre de globules blancs de 17 x10 3/μL, l'Hématocrite 37,9%; les Plaquettes de 84 x10 3/μL, le Taux de Prothrombine: 68%; le Temps de Céphaline Activé 27,7 sec, la Glycémie 3,65 g/L, la Bilirubine Totale 48 mg/L, la CRP 477,3 mg /L. Une TDM abdominale, (Figure 1) montrait des épanchements péritonéaux évoquant une péritonite aigue généralisée et des signes d'ischémie digestive en rapport avec une thrombose isolée de l'artère mésentérique supérieure très étendue et débutant à 2 cm de son ostium. L'aorte thoraco-abdominale et ses autres branches étaient saines sans lésions athéromateuses et il n'y avait pas de thrombus intra aortique. Un bilan cardiaque (ECG et ETT) s'avérait normal.

**Figure 2:**
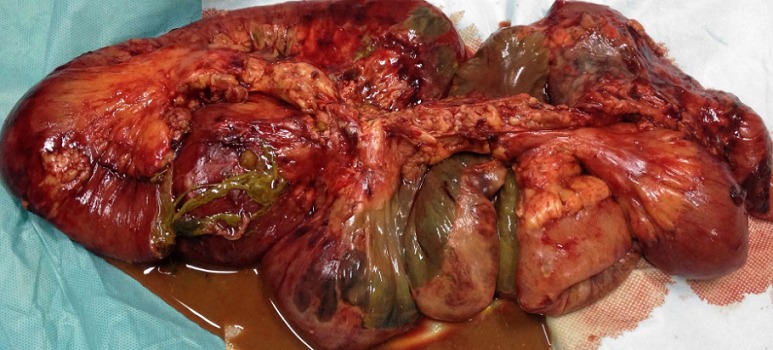
Pièce opératoire

**Figure 3:**
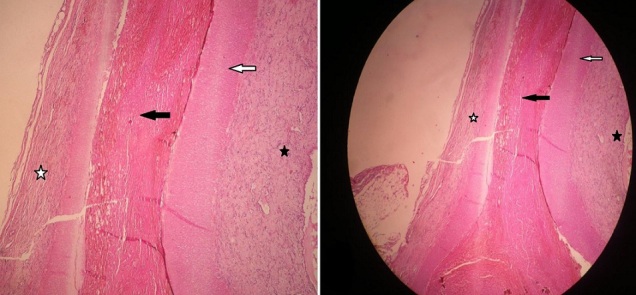
(anapathomopatologie) coloration Hématéine-Eosine (H&E) paroi artérielle de morphologie conservée (artère mésentérique supérieure). La lumière est obstruée par un thrombus (étoile noire). Absence de nécrose fibrinoide, la limitante élastique interne et la média sont saines (flèches blanche et noire). Absence de signe de vascularite. En périartérielle un discret infiltrat inflammatoire lymphoplasmocytaire (étoile blanche) A) H&E grossissement x 40; B) faible grossissement

Une laparotomie trouvait un épanchement purulent abondant sous méso colique, l'intestin grêle ischémié et perforé par endroits (Figure 2), 30 cm après l'angle duodéno jéjunale jusqu’à 40 cm en avant de la valvule iléo caecale de Bauhin. Il était réséqué en passant en zone saine, une toilette abdominale abondante était faite, et une double stomies réalisée. Des prélèvements de l'artère mésentérique supérieure et toute la pièce opératoire étaient effectués pour étude anatomopathologique. Il était transféré en réanimation chirurgicale ou charge il décédera 2 jours plus tard du fait de l’étendue de ses lésions et du délai de prise en charge. À l´examen histologique de la pièce opératoire, l'iléon reséqué présentait une muqueuse sévèrement ischémique avec des plages de nécrose. Il y avait une atteinte généralisée des vaisseaux mésentériques et sous muqueux. L'artère mésentérique supérieur et plusieurs artères de moyen calibre présentaient un aspect inflammatoire au niveau de l´intima et de l´adventice, épargnant de la couche musculaire (Figure 3). Il y avait une prolifération fibroblastique intimale marquée et la limitante élastique interne était intacte. L'infiltrat inflammatoire était constitué essentiellement de lymphocytes, histiocytes et éosinophiles, tandis qu'il y avait une absence de neutrophiles. Un thrombus riche en neutrophile était observé dans la lumière de l'artère mésentérique supérieure; mais il n´y n´avait aucun signe d´athérome, de calcification ou d'embole de cholestérol. Ces anomalies vasculaires faisaient penser à une vasculopathie primaire plus qu’à des modifications secondaires dues à une autre pathologie; elles étaient avec une atteinte des artères digestives due à la maladie de Buerger.

## Discussion

La Thrombo-angéite oblitérante (TAO, la maladie de Buerger) est une maladie relativement rare des vaisseaux sanguins. Elle concerne surtout les artères et veines de taille moyenne à petite des membres, et atteint classiquement les hommes jeunes, tabagiques chroniques [1, 2]. De nombreuses tentatives pour définir les critères diagnostiques de la TAO ont été réalisées [2, 3] et les plus utilisés actuellement sont les critères de Shionoya [4] (Tableau 1). La première description de participation mésentérique de cette maladie à été faite par Leo- Buerger en 1924 [5]. Une trentaine de cas ont par la suite été décrits [5–7]. Ils atteignaient principalement les hommes dont l’âge moyen était de 41,5 ans, bien que 2 cas de femmes aient été rapportés [5]. Les atteintes intestinales de la TAO peuvent être asymptomatiques (de découverte fortuite), aigues (découvertes au décours d'un épisode d'ischémie aigue mésentérique) ou chroniques (réalisant un tableau d'insuffisance entéro-mésentérique). La distribution des lésions est ubiquitaire, mais elle concerne principalement l'intestin grêle dans 50% des cas, le gros intestin dans 40%, les deux dans 10% des cas [5, 6]. Les lésions vasculaires microscopiques dans la maladie de Buerger se distinguent de celles de l´artériosclérose oblitérante par la présence de thrombus cellulaire, une média bien conservées, et une réaction inflammatoire au long de la paroi du vaisseau. Ces lésions décrites sur les vaisseaux périphériques, sont les mêmes retrouvées dans les rares cas d'atteintes viscérales [5, 6]. La TAO se distingue de la thrombose et de l'embolie artérielle ou veineuse simple par l´organisation cellulaire des thrombus, une réaction fibroblastique dans la média, et la prolifération endothéliale. Les lésions aiguës et anciennes peuvent être observées en microscopie. Ces modifications histopatologiques vasculaire dues à la maladie de Buerger sont les mêmes observées dans les artères ou les veines viscérales atteintes. La résection intestinale est la base du traitement chez les patients se présentant dans un tableau aigue, avec une mortalité qui reste très élevée [7, 8]. Quatre cas de revascularisations par pontage ont été rapportés chez des patients se présentant dans un tableau chronique avec une perméabilité primaire moyenne [6]. Souvent malheureusement, l’état du patient ou l’étendue des lésions imposent l´abstention thérapeutique. [8]

**Tableau 1:** Critères diagnostic de la maladie de Buerger; d'après Shigehiko Shionoya [4]

1	Antécédents de tabagisme
2	Apparition de symptômes avant l’âge de 50 ans
3	Occlusion des artères sous-poplitée
4	Participation du membre supérieur ou la phlébite migrante
5	Absence de facteurs de risque d'athérosclérose autres que le tabac

(Tous les critères doivent être présents)

## Conclusion

Les atteintes digestives de la maladie de Buerger sont exceptionnelles. La revascularisation des artères digestives atteintes, ne concerne que les cas ou l’évolution est chronique. L’éviction de l'intoxication tabagique associé la surveillance accrue des patients présentant cette maladie, sont les seuls moyens permettant d’éviter son évolution vers une atteinte digestive aigue souvent fatale.
